# One-pass selective conversion of syngas to *para*-xylene†
†Electronic supplementary information (ESI) available: Details in Experimental section and supporting figures and tables. See DOI: 10.1039/c7sc03427j


**DOI:** 10.1039/c7sc03427j

**Published:** 2017-10-16

**Authors:** Peipei Zhang, Li Tan, Guohui Yang, Noritatsu Tsubaki

**Affiliations:** a Department of Applied Chemistry , School of Engineering , University of Toyama , Gofuku 3190 , Toyama 930-8555 , Japan . Email: thomas@eng.u-toyama.ac.jp ; Email: tsubaki@eng.u-toyama.ac.jp

## Abstract

The finite petroleum resources and environmental crisis compel the development of non-petroleum carbon resources by chemical transformation routes. Syngas (CO + H_2_) is a crucial junction point that exclusively bridges the non-petroleum carbon resources and other basic chemicals like alcohols, alkane/alkenes, *etc.* However, the one-pass conversion of syngas to value-added aromatics, especially *para*-xylene, is still a big challenge. Here we presented a promising hybrid catalyst, named Cr/Zn–Zn/Z5@S1, to effectively realize the one-pass conversion of syngas to *para*-xylene. This hybrid catalyst exhibited enhanced activity on syngas conversion (CO conversion of 55.0%), good stability of catalyst lifetime and considerable selectivity of *para*-xylene (27.6% in the total products and 77.3% in xylene). The characterization and catalytic performance evaluation revealed that the well-designed core–shell Zn/Z5@S1 zeolite, as a vital part of this Cr/Zn–Zn/Z5@S1 hybrid catalyst, substantially contributed to its extreme performance for the *para*-xylene one-pass precise synthesis from syngas. The concerted combination of two components in this hybrid catalyst can effectively depress the formation of unwanted by-products and facilitate the oriented synthesis of *para*-xylene from syngas with unprecedented efficiency at the same time.

## Abstract 2

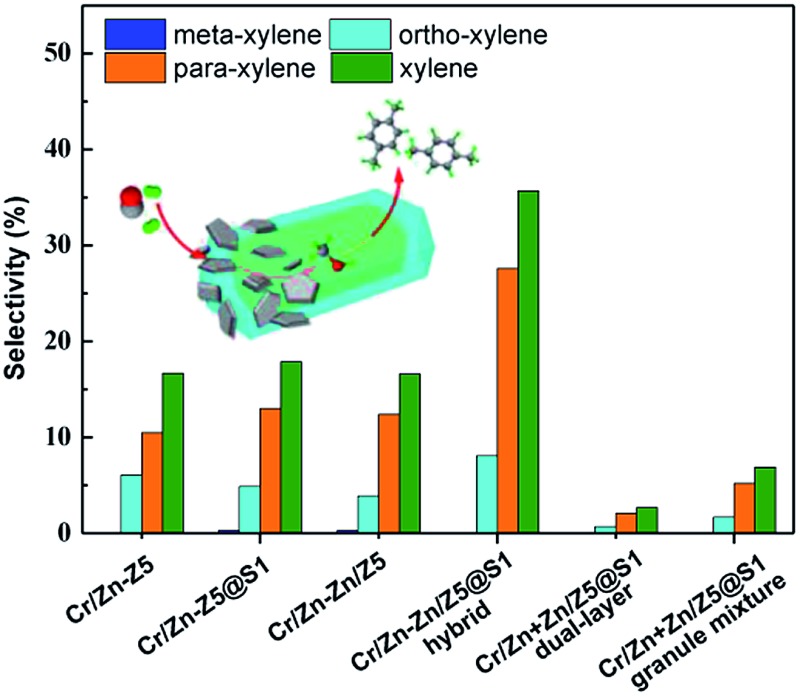
We presented a promising hybrid catalyst, named Cr/Zn–Zn/Z5@S1, to effectively realize the one-pass selective conversion of syngas to *para*-xylene.

## Introduction

Syngas, a mixture gas of CO and H_2_, can be produced from non-petroleum sources, including natural gas, coal and biomass, *etc.* It is usually used as the raw material to produce other chemicals like dimethyl ether, light olefins, gasoline, diesel, and even aromatics through the indirect conversion process of Fischer–Tropsch synthesis (FTS)/methanol synthesis and other subsequent catalytic reactions.^1^–^10^ The production of alkanes/alkenes and aromatics from methanol has received much attention in recent years.^11^–^14^ But the methanol as an intermediate is synthesized separately from syngas through a methanol synthesis reaction.^15^ To date, the one-pass selective conversion of syngas into the target products, like dimethyl ether, isoparaffins/olefins and aromatics has made noticeable progress,^16^–^21^ although the catalyst activity and CO_2_ formation are still a challenge. Aromatic hydrocarbons, generally being produced through the petroleum industry, are one of the most important basic chemicals. The production of aromatic hydrocarbons from syngas is a crucial alternative process. Among all kinds of aromatic hydrocarbons, *para*-xylene (PX) is a significant value-added chemical, since it has an important use in producing terephthalic acid (TPA) and other chemicals.^22^ The PX is produced by the catalytic reforming of petroleum naphtha as part of the BTX aromatics (benzene, toluene and xylene isomers).^23^ And the separation of PX from aromatic mixtures is a high energy-consuming process. Alternative technology for the one-pass synthesis of PX based on a non-petroleum route is required due to the growing demand for PX annually and decreasing reserves of petroleum.

Aromatic synthesis from methanol has been widely studied.^24^,^25^ A higher PX selectivity is always desired in the methanol to aromatics (MTA) reactions.^26^,^27^ H-ZSM-5 zeolite is known for converting methanol to PX because of its channel architecture with suitable size and alterable acidity. Unfortunately, the isomerization of xylene, the main side reaction, simultaneously occurs on the external surface of the H-ZSM-5 zeolite leading to low PX selectivity.^26^ Several approaches, such as metal or metal oxide impregnation, non-acidic silica deposition and so on, have been conducted to passivate the exterior acid sites of H-ZSM-5 to avoid the undesirable xylene isomerization.^28^–^35^

The methanol used for the MTA reaction must be synthesized separately from the syngas. The direct synthesis of aromatics from syngas should be more favourable in view of thermodynamics and economics. However, previous studies on the direct conversion of syngas to alkanes/alkenes only reported the formation of an aromatic mixture, never xylene.^36^,^37^ The direct conversion of syngas to aromatics is a complicated process over a typical catalyst mixture. Generally, methanol is first synthesized from syngas over a methanol synthesis catalyst, and then the formed methanol will be converted to the aromatics over an acidic zeolite catalyst. Due to the complex reaction paths and harsh reaction conditions, the selectivity of xylene, let alone PX, among the generated aromatics is very low. Designing an efficient route to directly convert syngas into PX is highly required, but it is still a serious challenge and, until now, there have been no reports about the one-pass selective conversion of syngas into PX.

**Fig. 1:**
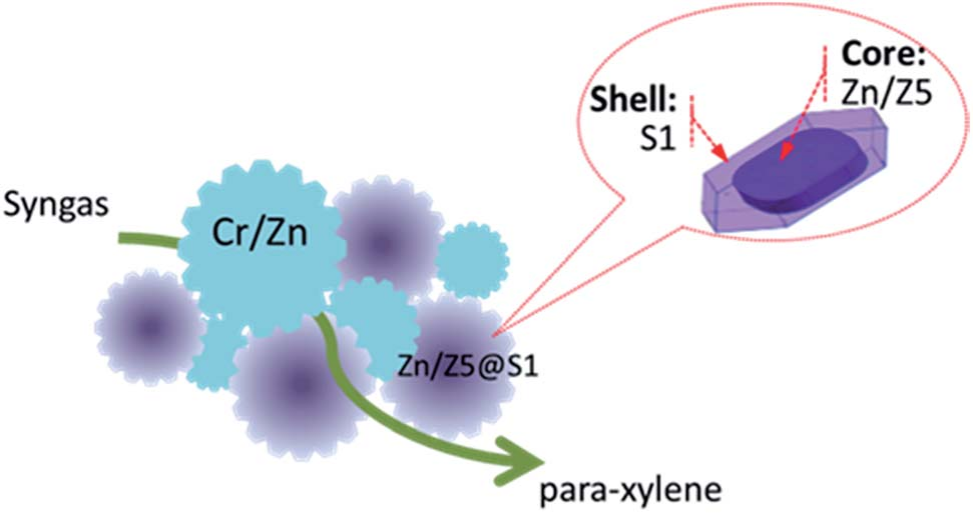
Illustration of the one-pass selective conversion of syngas to *para*-xylene over the designed hybrid catalyst Cr/Zn–Zn/Z5@S1.

In this report, we will present a hybrid catalyst that contains a Cr-based catalyst and core–shell-structured zeolite. With this novel hybrid catalyst we can facilely realize the one-pass selective conversion of syngas to PX. Typically, the hybrid catalyst, named Cr/Zn–Zn/Z5@S1, is composed of two components: one is Cr/Zn and the other is a core–shell-structured zeolite, Zn/Z5@S1 (zinc doped H-ZSM-5 single crystal zeolite encapsulated with one silicalite-1 zeolite shell). The Cr/Zn component with a ZnCr_2_O_4_ spinel structure is prepared through a co-precipitation method. The core–shell-structured Zn/Z5@S1 zeolite with a non-acidic silicalite-1 shell is synthesized through hydrothermal synthesis. All the information about this Cr/Zn–Zn/Z5@S1 hybrid catalyst preparation has been described in detail in the ESI.† As illustrated by the demo route in Fig. 1, this hybrid catalyst Cr/Zn–Zn/Z5@S1 will realize the highly selective synthesis of PX directly from syngas.

## Results and discussion

The detailed physical properties of the Cr/Zn component are characterized by XRD, SEM, XPS and H_2_-TPR (Fig. S1–S4†). The analysis results indicate that the ZnCr_2_O_4_ spinel is successfully synthesized on the Cr/Zn component. The synthesis procedure for the core–shell-structured Z5@S1 and Zn/Z5@S1 zeolite components is shown in Scheme S1.† The detailed preparation methods of these zeolite components are also described in the Experimental section of the ESI.† The original Z5 (H-ZSM-5, Si/Al = 46) or Zn/Z5 (Zn doped Z5, 1.0 wt% by ICP analysis) are used as the zeolite cores for the core–shell-structured Z5@S1 and Zn/Z5@S1 zeolite syntheses, respectively, by epitaxially growing a silicalite-1 shell on the zeolite cores. The XRD profiles of all of the zeolite components used for the hybrid catalyst preparation are also shown in Fig. S1.† The classic peaks of the MFI zeolite appear in the ranges of 2*θ* = 7–10° and 22–26°, indicating that the MFI structure of the Z5 zeolite is stable after the modification by Zn and the encapsulation with the silicalite-1 zeolite shell.^38^ In addition, no peaks belonging to the Zn species are discovered on the Zn/Z5 and Zn/Z5@S1 zeolites because the amount of Zn on these samples is too low to be detected.

**Fig. 2:**
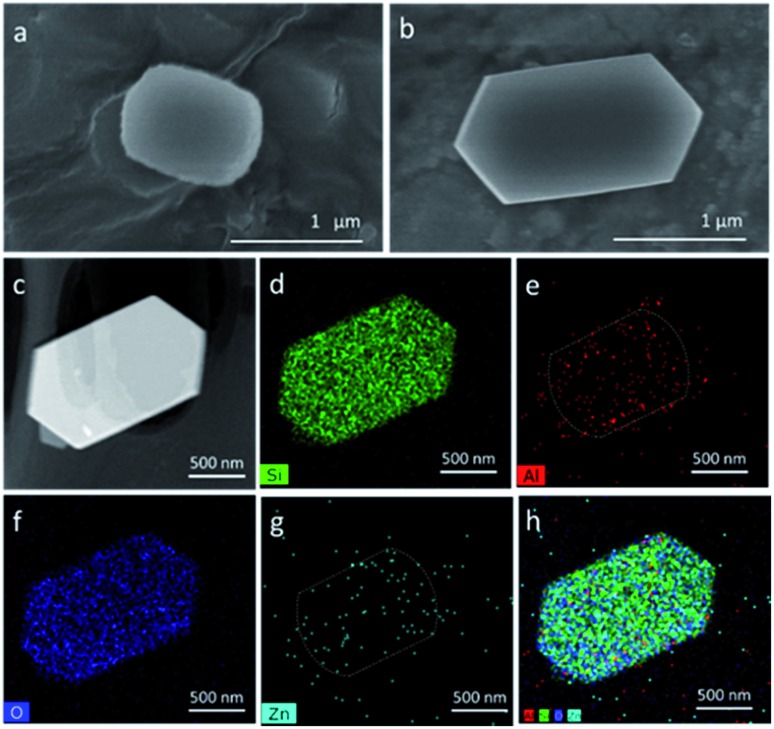
FE-SEM images of (a) Zn/Z5 and (b) the core–shell-structured Zn/Z5@S1 zeolite components; (c) STEM image of the Zn/Z5@S1 zeolite component and the corresponding STEM EDS mapping of (d) Si, (e) Al, (f) O, (g) Zn and (h) the combined Si, Al, O and Zn.

We employed SEM to characterize all the zeolite components and the results are shown in Fig. S5.† The surface morphology of the typical Zn/Z5 and Zn/Z5@S1 zeolites measured by FE-SEM is given in Fig. 2a and b. The Zn doped Z5 zeolite will not affect the original zeolite morphology, as indicated by Zn/Z5 zeolite in Fig. 2a and S5c.† With Z5 or Zn/Z5 as the mother zeolite, we epitaxially grow their bodies through a hydrothermal synthesis process in the synthesis solution for silicalite-1 zeolite growth, in order to get the silicalite-1 shell encapsulated zeolite Z5@S1 and Zn/Z5@S1. For the core–shell-structured Zn/Z5@S1 zeolite, as shown in Fig. 2b, the Zn/Z5 core zeolite has been fully encapsulated by the silicalite-1 zeolite layer. The Zn/Z5@S1 sample has a hexagonal exterior shape with a particle length of around 2.5 μm. The original Zn/Z5 core zeolite has a rounded hexagon shape with the smaller size of around 1 μm (Fig. 2a). The epitaxial growth of the silicalite-1 shell is along the outside surface of the Z5 crystal.^15^,^34^ STEM and EDS mapping analysis on the core–shell-structured Zn/Z5@S1 zeolite are given in Fig. 2c–h. The Al and Zn elements are distributed mainly inside the white circle, indicating the Zn/Z5 core section of the Zn/Z5@S1 zeolite. Moreover, XPS analysis also shows that the number of Zn and Al species is zero in the near-surface regions of the Zn/Z5@S1 zeolite (Fig. S6 and Table S1†). The physical properties of all of the Cr/Zn and zeolite components are also summarized in Table S2 and Fig. S7.† All of the analysis results prove that the prepared Zn/Z5@S1 zeolite possesses an ideal core–shell structure, in which the Zn/Z5 zeolite core is entirely enwrapped by the silicalite-1 zeolite shell.

The acidity of all zeolite components for hybrid catalyst preparation was evaluated by NH_3_-TPD, and the results are shown in Tables S3 and S4, and in Fig. S8 and S9.† The type of acidic sites for Z5 and Zn/Z5 are similar, while the acidic concentration of Zn/Z5 is less than that of Z5, because a few Al atoms in Z5 are replaced by Zn. The core–shell-structured Z5@S1 and Zn/Z5@S1 have a lower acidic strength and acidic concentration than those of Z5 and Zn/Z5, indicating that the introduced silicalite-1 shell neutralizes and seals the surface acidic sites of Z5. The designed silicalite-1 shell will effectively seal the surface acidic sites of the core zeolite, depressing the formation of *ortho*-, *meta*-xylene, at the same time as boosting the generation of PX in the following reaction.^27^,^39^

**Fig. 3:**
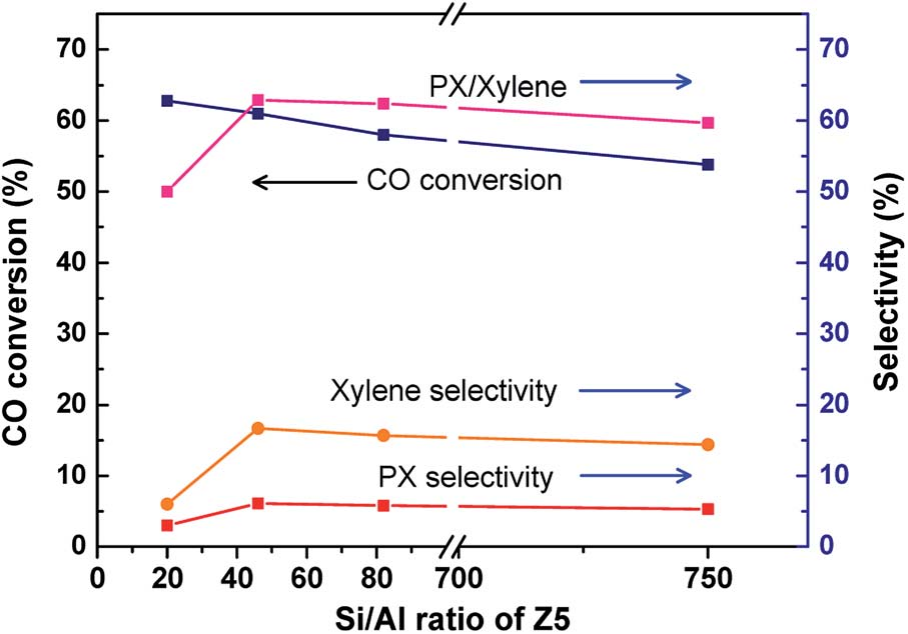
The effect of a varied Si/Al ratio of Z5 in the simplest hybrid catalyst Cr/Zn–Z5 for the one-pass selective conversion of syngas to PX.

By physically mixing the Cr/Zn component and zeolite components (Z5, Zn/Z5, Z5@S1 and Zn/Z5@S1), we get a series of hybrid catalysts Cr/Zn–Z5, Cr/Zn–Zn/Z5, Cr/Zn–Z5@S1 and Cr/Zn–Zn/Z5@S1. The effect of a varied Si/Al ratio (20, 46, 82 and 750) of Z5 in the simplest hybrid catalyst Cr/Zn–Z5 for PX one-pass synthesis from syngas is investigated, as shown in Fig. 3 and Table S5.† It is clear that the most suitable Si/Al ratio of Z5 is around 46. Therefore, the following hybrid catalysts are prepared with Z5 (Si/Al = 46) as the zeolite component.

All of the hybrid catalysts are evaluated to investigate their catalytic performance for the one-pass PX synthesis from syngas, and the reaction results are compared in Table 1. The catalytic reaction is conducted under a pressure of 5.0 MPa and a temperature of 673 K. The single Cr/Zn catalyst, as a reference catalyst, is also tested under the same reaction conditions. The CO conversion over the Cr/Zn catalyst is 27.5% with the major products of MeOH, DME and CH_4_ (Table S6†). In contrast, all of the hybrid catalysts present higher CO conversion and zero formation of methanol and DME. The introduced zeolite components in the hybrid catalysts promote CO conversion by shifting the thermodynamic equilibrium of MeOH synthesis. In addition to the enhanced CO conversion, all of the hybrid catalysts can convert syngas directly into alkanes/alkenes and xylene. As given in Table 1, the Cr/Zn–Zn/Z5@S1 hybrid catalyst shows excellent xylene selectivity of 35.7%, in which the selectivity of PX reaches up to 27.6%. The PX selectivity in the total xylene is 77.3%, the highest value among all of the tested catalysts.

**Table 1:** One-pass selective conversion of syngas to PX over the hybrid catalysts^*a*^

Catalysts	Conv. (%)	Selectivity^*b*^ (%)						
	CO	MeOH + DME	CH_4_	C_2_–C_5_	OX	MX	PX	PX/X^*c*^ (%)
Cr/Zn	27.5	54.1	30.4	14.0	0	0	0	—
Cr/Zn–Z5	61.0	0	2.4	41.4	6.1	0.1	10.5	62.9
Cr/Zn–Z5@S1	55.2	0	2.4	44.5	4.9	0.3	13.0	71.4
Cr/Zn–Zn/Z5	66.4	0	3.2	38.5	3.9	0.3	12.4	74.7
Cr/Zn–Zn/Z5@S1 hybrid	55.0	0	4.4	33.6	8.1	0	27.6	77.3
Cr/Zn + Zn/Z5@S1 dual-layer	34.5	0.3	26.8	59.1	0.7	0	2.1	75.0
Cr/Zn + Zn/Z5@S1 granule mixture	50.4	0.1	7.2	66.6	1.7	0	5.2	75.4

^*a*^Reaction conditions: 5.0 MPa, 673 K, W/F = 20.7 g h mol^–1^, syngas : H_2_/CO = 2.1, 4 h, hybrid catalysts 0.5 g (Cr/Zn : zeolite = 2).

^*b*^The selectivity of C_2_–C_5_, OX, MX, and PX in all of the products, C_2_–C_5_ including paraffins and olefins, OX = *ortho*-xylene, MX = *meta*-xylene, PX = *para*-xylene.

^*c*^PX/X: the ratio of PX to all xylene.

**Fig. 4:**
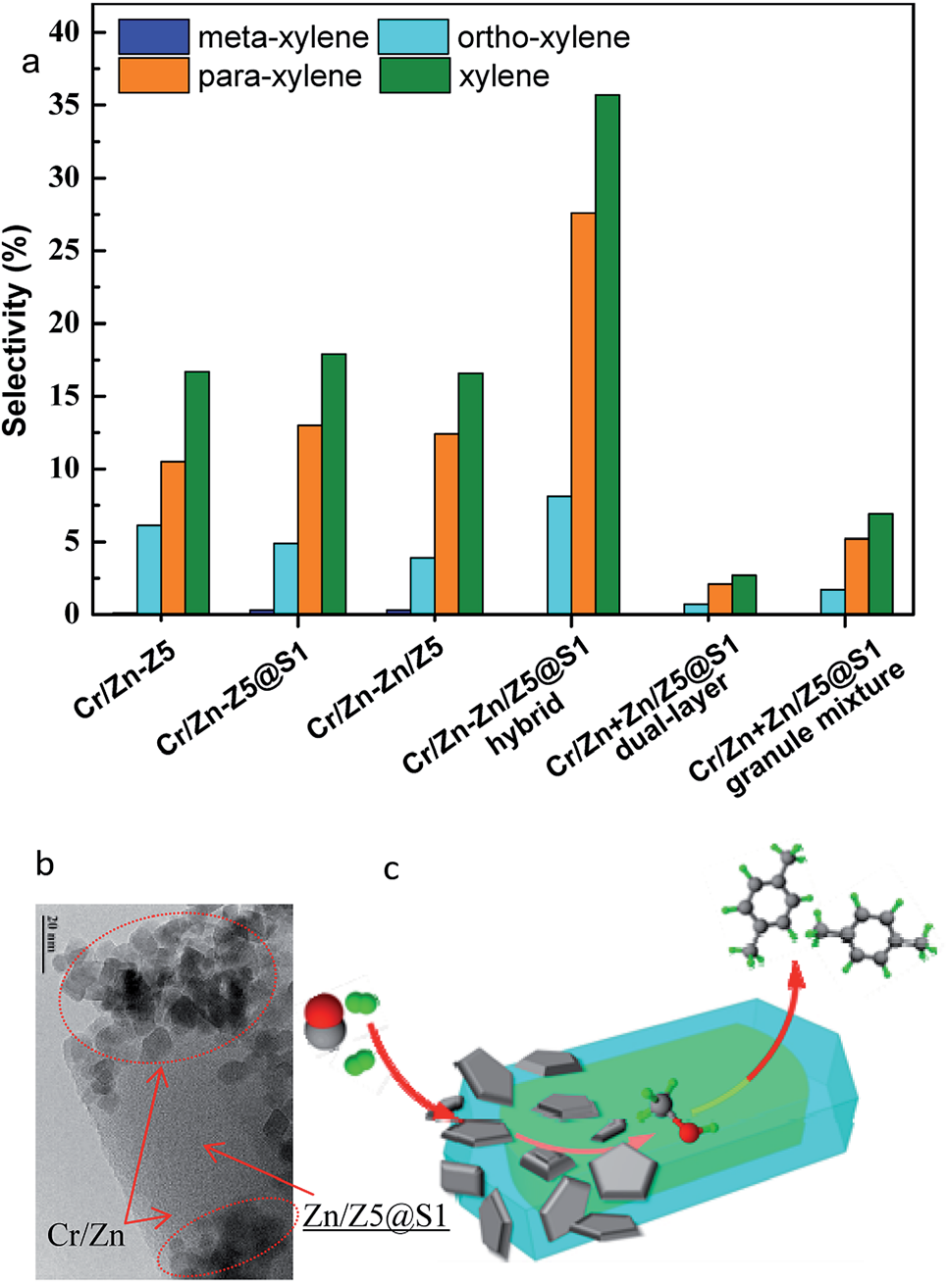
(a) The distribution of total xylene and the isomers (OX, MX and PX) over various catalysts; (b) TEM image of the Cr/Zn–Zn/Z5@S1 hybrid catalyst; (c) the reaction mechanism of the one-pass selective syngas conversion to *para*-xylene over the hybrid catalyst Cr/Zn–Zn/Z5@S1.

From the view point of diffusion, the distance between the components of Cr/Zn and Zn/Z5@S1 is considerably crucial for the hybrid catalyst. Other component assembly models of Cr/Zn + Zn/Z5@S1 (dual-layer) and Cr/Zn + Zn/Z5@S1 (granule mixture), as the references for the Cr/Zn–Zn/Z5@S1 hybrid catalyst were also designed and evaluated (Tables 1 and S6, Fig. 4a and S10†). The Cr/Zn + Zn/Z5@S1 (dual-layer) comprises a top Cr/Zn layer and a bottom Zn/Z5@S1 layer in one reactor (Fig. S10b†). The formed MeOH and DME in the top Cr/Zn layer are largely converted into alkanes/alkenes (C_2_–C_5_ = 59.1%), but little PX (2.1%). Methane formation (26.8%) cannot be inhibited, because of the long distance between Cr/Zn and Zn/Z5@S1. The Cr/Zn + Zn/Z5@S1 (granule mixture) can further decrease the distance between Cr/Zn and Zn/Z5@S1 (Fig. S10c†). The CO conversion is promoted up to 50.4%, obviously higher than that of the single Cr/Zn and Cr/Zn + Zn/Z5@S1 (dual-layer). Methane selectivity is only 7.2%, but alkanes/alkenes (C_2_–C_5_ = 66.6%) existed still, with a low PX selectivity of 5.2%. In contrast, the Cr/Zn–Zn/Z5@S1 hybrid catalyst, prepared by milling two component powders adequately, ensures the shortest distance between the components in the hybrid catalyst (Fig. S10d†). As a result, we got the highest CO conversion (55.0%) and the highest PX (27.6%) selectivity, at the same time as a lower selectivity of CH_4_ and C_2_–C_5_, as given in Table 1. The two components in the hybrid catalyst are close to each other. The formed intermediates can diffuse quickly to the Zn/Z5@S1 component, and are converted into PX. Besides, rapidly removing the intermediates from the Cr/Zn component can push the thermodynamic equilibrium to shift towards methanol synthesis from syngas, therefore depressing other competitive side reactions like methanation, *etc.* The comparison of the three catalyst models, the Cr/Zn–Zn/Z5@S1 hybrid catalyst, Cr/Zn + Zn/Z5@S1 (granule mixture) and Cr/Zn + Zn/Z5@S1 (dual-layer), proves that a short distance between the two components is required for the design of a hybrid catalyst to realize PX selective synthesis directly from syngas.

The selectivity of xylene and PX over the hybrid catalysts is also compared in Fig. 4a. Both of the highest selectivities for xylene (35.7%) and PX (27.6%) were successfully realized on the Cr/Zn–Zn/Z5@S1 hybrid catalyst. The formation of *meta*-xylene has been completely suppressed, and the major by-product in the xylene isomers is only *ortho*-xylene. The time on stream of xylene and PX selectivity in the xylene isomers is shown in Fig. S11.† The Cr/Zn–Zn/Z5@S1 hybrid catalyst demonstrates enhanced selectivity and excellent stability for PX synthesis during the total reaction time. As indicated by the TEM image in Fig. 4b, the Cr/Zn–Zn/Z5@S1 hybrid catalyst is composed of a Cr/Zn component and a core–shell-structured Zn/Z5@S1 zeolite component. In the reaction (as illustrated by Fig. 4c), syngas is first converted into methanol on the Cr/Zn component, and then the formed methanol *in situ* undergoes a series of reaction steps such as dehydration, C–C bond coupling, *etc.*, to generate PX over the core–shell-structured Zn/Z5@S1 zeolite component. The two components in this Cr/Zn–Zn/Z5@S1 hybrid catalyst contact tightly, cooperate concertedly and promote mutually. The highest PX selectivity among the products obtained by the Cr/Zn–Zn/Z5@S1 hybrid catalyst should be attributed to its zeolite component of Zn/Z5@S1 with a special core–shell structure. The ion-exchange of the Z5 zeolite with Zn generates Lewis acid sites, replacing the previous strong acid sites, hence the aromatic selectivity of the hybrid catalysts is clearly improved.^40^ The silicalite-1 shell can seal the exposed external acidic sites of the Zn/Z5 core zeolite, in order to depress xylene isomerization. As a result, the Cr/Zn–Zn/Z5@S1 hybrid catalyst successfully converts syngas into PX with extremely higher selectivity. In addition, varied weight ratios of Cr/Zn to Zn/Z5@S1 in this hybrid catalyst clearly affect its catalytic performance (Table S7†). The more suitable weight ratio of the two components in this Cr/Zn–Zn/Z5@S1 hybrid catalyst is 2 : 1.

Carbon deposition on the zeolite catalyst is the major factor that will affect the catalyst’s life time and performance. TG is employed to measure the carbonaceous species deposited on the spent hybrid catalysts (after 10 h reaction) and the result is given in Fig. S12.† The coke deposition rate of Cr/Zn–Zn/Z5@S1 is in last place among all the catalysts. The coke formation on the zeolite catalyst is initiated at the edge of straight pores in contact with the crystal outer surface.^41^ However, for the Cr/Zn–Zn/Z5@S1 hybrid catalyst, the silicalite-1 shell is epitaxially synthesized at the surface of the Zn/Z5 zeolite, which leads to a direct pore to pore connection between Z5 and silicalite-1 during the hydrothermal synthesis process.^42^,^43^ Therefore, it is demonstrated that the coke formation on the Cr/Zn–Zn/Z5@S1 hybrid catalyst is effectively depressed, ensuring its substantial stability among all the tested catalysts.

For the prospective further enhancement of the xylene yield and *para*-xylene selectivity, it is practical to tune syngas composition and/or the thickness of silicalite-1 zeolite shell enwrapping the single-crystal Zn/Z5 core zeolite of the hybrid catalyst, in order to further depress the formation of alkanes/alkenes and other aromatic by-products. Furthermore, precisely controlling the crystal size or channel properties of the Zn/Z5 core zeolite can further enhance the *para*-xylene selectivity. At the present stage, *meta*-xylene formation has been completely stopped. Considering the similar kinetic diameters of *ortho*-xylene and *meta*-xylene, which are larger than that of *para*-xylene, further modifying the channels and adjusting the crystal size of the Zn/Z5 core zeolite should be effective tools to tune the intracrystalline diffusivity of products, therefore erasing the formation of *ortho*-xylene, and simultaneously lowering the selectivities of other hydrocarbon by-products.

## Conclusions

In summary, we presented a successful hybrid catalyst Cr/Zn–Zn/Z5@S1 for the one-pass conversion of syngas to *para*-xylene with high activity, selectivity, stability and lower CO_2_ formation. The selectivity of PX obtained by this hybrid catalyst reached up to 77.3% in the xylene isomers and accounted for 27.6% of the total hydrocarbon products at the CO conversion of 55.0%. This hybrid catalyst contained two components: one was Cr/Zn and the other one was a core–shell-structured Zn/Z5@S1 zeolite. The combination of two components in this hybrid catalyst enabled a well-organized tandem catalysis process, performing syngas to methanol and methanol to PX exclusively. The special core–shell structure of Zn/Z5@S1 could effectively seal the exposed active sites of the zeolite, in order to depress the formation of unwanted xylene isomers and promote the oriented synthesis of PX. The hybrid catalyst Cr/Zn–Zn/Z5@S1 reported in this paper is extremely promising as an industrial catalyst, not only for PX synthesis, but also for the direct conversion of syngas to value-added chemicals.

## Conflicts of interest

There are no conflicts to declare.

## Supplementary Material

Supplementary information
