# Supplementary material for: One-pass selective conversion of syngas to para-xylene
Source: Chem Sci. 2017 Oct 16;8(12):7941–6. doi: 10.1039/c7sc03427j (PMC5858746; doi:10.1039/c7sc03427j)
Supplement: Supplementary file 1 [file SC-008-C7SC03427J-s001.pdf]

Electronic Supplementary Material (ESI) for Chemical Science.
This journal is © The Royal Society of Chemistry 2017

Supporting Information

One-pass Selective Conversion of Syngas to *para*-Xylene

Peipei Zhang,[+] Li Tan,[+] Guohui Yang,* and Noritatsu Tsubaki*

Applied Chemistry, School of Engineering, University of Toyama, Gofuku 3190, Toyama 930-8555, Japan.

E-mail address: thomas@eng.u-toyama.ac.jp, tsubaki@eng.u-toyama.ac.jp

Table of Contents

Experimental section S3-7

Figures and Tables

Scheme S1. Schematic illustration S8

Figure S1. XRD images S9

Figure S2. SEM images of Cr/Zn S10

Figure S3. XPS spectra of Cr/Zn S11

Figure S4. H_2-TPR profile of Cr/Zn S12

Figure S5. SEM images of Z5, Zn/Z5, Z5@S1 and Zn/Z5@S1 zeolite S13

Figure S6. XPS curves of Z5, Zn/Z5, Z5@S1 and Zn/Z5@S1 zeolite S14

Figure S7. N_2 adsorption-desorption isotherms S15

Figure S8. NH_3-TPD images of Z5, Zn/Z5, Z5@S1 and Zn/Z5@S1 zeolite S16

Figure S9. NH_3-TPD curves of Z5 with different of Si/Al ratio S17

Figure S10. The effect of varied assembly models of two active components on the catalytic performance in reaction S18

Figure S11. Time on stream of xylene and p-xylene selectivity S19

Figure S12. TG data S20

Table S1. XPS analysis results S21

Table S2. BET data

S22

Table S3. NH_3-TPD data of Z5, Zn/Z5, Z5@S1 and Zn/Z5@S1 zeolite S23

Table S4. NH_3-TPD data of Z5 with varied Si/Al ratio S24

Table S5. Catalytic performance of Cr/Zn-Z5 with varied Si/Al ratio S25

Table S6. Catalytic performance of varied hybrid catalysts S26

Table S7. Catalytic performance of Cr/Zn-Zn/Z5@S1 with varied weight ratio of Cr/Zn to Zn/Z5@S1 S27

Experimental section

1 Catalysts preparation

1.1. Cr/Zn catalyst preparation

The Cr/Zn catalyst was prepared with the Cr to Zn atomic ratio of 2/1 through a typical co-precipitation method. $Zn(NO_3)_2{\cdot}6H_2O$ and $Cr(NO_3)_3{\cdot}9H_2O$ were first dissolved in deionized water; the above solution and $(NH_4)_2CO_3$ aqueous solution were added dividedly into one beaker by dropwise with constant pH of 7~8 at 343 K under continuous stirring for 1 h, then followed by 3 h aging time at the same temperature. Finally, the obtained mixture was filtered and washed by deionized water, followed by drying in an oven at 393 K overnight and by calcination in air at 773 K for 3 h. The final sample was granulated into a size of 0.85-1.70 mm and denoted as Cr/Zn.

1.2. H-ZSM-5 (Z5) zeolite preparation

H-ZSM-5 zeolite with different Si/Al ratio (20, 46, 82 and 750) was prepared by hydrothermal synthesis method through adjusting the Si/Al ratios of precursor. The synthesis solution of H-ZSM-5 (2TEOS: 0.68TPAOH: 8EtOH: $120H_2O$: xAl_2O_3, x=0.001-0.05) was enclosed in an autoclave and heated to 453 K for 24 h with a rotation rate of 2 r/min. After crystallization, the samples were separated from mother solution and washed by deionized water for several times until the pH=7, then followed by drying at 393 K for 12 h. Finally, the H-ZSM-5 zeolite was obtained after calcination at 823 K for 6 h with heating rate of 1 K/min, and denoted as Z5 in this paper.

1.3. The Zn/Z5 catalyst preparation

The obtained Z5 (Si/Al=46) sample was modified by Zn through ion-exchange method. Briefly, 1.5 g Z5 powder was added to 0.1 M $Zn(NO_3)_2$ aqueous solution at 353 K with continuously stirring for 15 h. After stirring, the obtained powder was washed by deionized water for three times, followed by drying at 393 K and calcination at 823 K for 5 h. The prepared sample was denoted as Zn/Z5.

1.4. The preparation of core-shell-structured Zn/Z5@S1 and Z5@S1 zeolite

The epitaxial crystal growth of Zn/Z5 (Si/Al=46) or Z5 (Si/Al=46) was performed through hydrothermal synthesis in the mother liquid for Silicalite-1 zeolite growth. 1.0 g Zn/Z5 or Z5 powder was added to the zeolite synthesis solution with the recipe of 1.00SiO_2: 0.06TPAOH: 16.0EtOH: 240H_2O. Then the mixture was moved into a teflon-lined autoclave for zeolite crystal epitaxial growth at 453 K for 24 h with a rotation rate of 3 r/min. The sample collection process is similar to that of Z5 sample. The above Silicalite-1 shell coating process was conducted twice. The obtained samples were named as Zn/Z5@S1 and Z5@S1, respectively.

1.5. The hybrid catalysts preparation

All the hybrid catalysts were prepared by physically mixing the two components of Cr/Zn and Z5-based zeolite, followed by grinding and granulating into the required size in the range of 0.85-1.70 mm. In order to investigate the effect of Si/Al ratio of Z5 on the hybrid catalyst performance, we first prepared a series of simplest hybrid catalyst Cr/Zn-Z5 with different Z5 (Si/Al=20, 46, 82 and 750) as the zeolite components. The Z5 zeolite (Si/Al=46) derived zeolite components, Zn/Z5, Z5@S1 and Zn/Z5@S1, were used for the preparation of hybrid catalysts Cr/Zn-Zn/Z5, Cr/Zn-Z5@S1 and Cr/Zn-Zn/Z5@S1.

1.6. The dual-layer catalyst model in reactor

As the reference of hybrid catalysts, the dual-layer catalyst model that was composed of top Cr/Zn layer and bottom Zn/Z5@S1 layer, named Cr/Zn+Zn/Z5@S1 (dual-layer), in the reactor had also been evaluated under the same reaction conditions. The Cr/Zn and Zn/Z5@S1 components were pelleted into the size of 0.85-1.70 mm respectively in advance.

1.7. The granule mixture catalyst model in reactor

As another reference catalyst of hybrid catalyst, the granular mixture catalyst model prepared by simply mixing granular Cr/Zn (0.8-1.70 mm) and granular Zn/Z5@S1 (0.8-1.7mm) had also been evaluated under the same reaction conditions. It was called Cr/Zn+Zn/Z5@S1 (granule mixture) in the followed discussion.

2. Catalysts characterization

The crystal structures of catalysts were determined by powder X-ray diffraction patterns (XRD), which were performed at Rigaku RINT 2400 X-ray Diffractometer equipped with a Cu Kα radiation. Scans were recorded in the 2θ range 5-90° with a step size of 0.02 °/s. The physical properties of catalysts, such as surface area, microspore volume and average pore size, were analyzed by N_2 physical adsorption at 77 K. These data were measured using Micrsmertics 3Flex specific surface area and porous physical adsorption analyzer. All samples were degassed in vacuum at 473 K for 5 h before measurement.

The scanning transmission electron microscopy images (STEM) and transmission electron microscopy images (TEM) were performed on a JEM-2100F (JEOL) at an acceleration voltage of 200 kV. The surface morphology and elemental composition were obtained by field-emission scanning electron microscope (FE-SEM, JEOL JSM-6700F) and scanning electron microscope (SEM, JEOL JSM-6360LV) with JED-2300 energy dispersive spectroscopy (EDS) attachment. The samples were per-treated in vacuum at 343 K overnight and sprayed platinum at 10 kv for 200 s before examination.

The X-ray photoelectron spectroscopy (XPS) analysis was done by Thermo Fisher Scientific ESCALAB 250Xi multifunctional X-ray photoelectron spectroscope, by which to determine the existence of Al and Zn on the outward appearance of catalysts. The data processing was performed using Avantage software. The inductive coupled plasma atomic emission spectrometry (ICP-AES) was employed to determine the real Zn content of zeolite materials using Perkin Elmer Optima 7300DV atomic emission spectrometry spectrometer.

NH_3 temperature programmed desorption (NH_3-TPD) was conducted by a catalyst analyzer BELCAT-B-TT (BEL Japan Co. Ltd.) equipped with a thermal conductivity detector (TCD). 0.03 g sample was pretreated at 423 K in He flow for 1 h, then 5% NH_3 in He was introduced for 1 h with flow rate of 20 ml/min while temperature was cooling down to 353 K. Subsequently, the microreactor was purified with pure He for 1 h. Finally, the NH_3 desorption procedure was proceeded in He flow (30 ml/min) by increasing the temperature from room temperature to 1073 K with a ramping rate of 5 K/min. The density of acid sites was quantified by peak area of the high-temperature NH_3-desorption peaks.

H_2 temperature programmed reduction (H_2-TPR) profiles of the samples were tested by a catalyst analyzer BELCAT-B-TT (BEL Japan Co. Ltd.) with a thermal conductivity detector (TCD). Cr/Zn catalysts (0.030 g) were pretreated at 423 K in He flow for 1 h, followed by temperature programmed reduction in H_2 with flow rate of 30 ml/min from room temperature to 1173 K with a ramping rate of 10 K/min.

Thermogravimetry (TG) measurement was carried out over 10 mg spent catalysts in air with flow rate of 30 ml/min using TA-60WS thermal analyzer (Shimadzu). The program was performed at the heating rate of 10 K/min from room temperature to 1173 K.

3. Catalysts evaluation for para-xylene (PX) direct synthesis from syngas

The catalyst performance evaluation of catalysts for PX direct synthesis from syngas was carried out in a fixed-bed steel reactor with internal diameter of 6 mm. All of catalysts were first reduced in the reactor for 10 h at 673 K with pure H_2. After cooling down to the room temperature, syngas with H_2/CO molar ratio of 2 was introduced into the reactor. Then, the reaction was conducted at 673 K under 5.0 MPa. All the products were kept in the gas phase from reactor to outlet and analyzed by an on-line gas chromatograph (GC-8A, Shimadzu), which was equipped with Plot S columns for the thermal conductivity detector (TCD), and HP-PLOT/Q columns for the flame ionization detector (FID). The CO conversion and the hydrocarbons selectivity were calculated with the followed equations, using Ar as an inner standard.

(1) $C_{CO}=(X_{CO,\ in} - X_{CO,\ out})/ (X_{CO,\ in}) \times 100\%$

C_{CO}: Conversion of CO, %; $X_{CO,\ in}$: Mole fraction of CO in pristine syngas;

$X_{CO,\ out}$: Mole fraction of CO in exit gas;

(2) $C_{CO2}=(X_{CO2,\ in}- X_{CO2,\ out})/ (X_{CO2,\ in}) \times 100\%$

C_{CO2}: Conversion of CO_2, %; $X_{CO2,\ in}$: Mole fraction of CO_2 in pristine syngas;

$X_{CO2,\ out}$: Mole fraction of CO_2 in exit gas;

(3) $$\mathbf{Sel_i} = \frac{\mathbf{R_{i,s} f_{i,m}}}{\sum_{\mathbf{i}} \mathbf{R_{i,s} f_{i,m}}}$$

Sel_i: Mass selectivity of component i, %; $f_{i,\ m}$: Factor of mass correction;

$R_{i,\ s}$: Area ratio of hydrocarbon in chromatogram.

(4) $$\mathbf{PX/X} = \frac{\mathbf{Sel_{PX}}}{\mathbf{Sel_{OX}} + \mathbf{Sel_{MX}} + \mathbf{Sel_{PX}}} \times 100\%$$

$^{PX}/_{X}$: Selectivity of para-xylene in total xylene isomers;

Sel_{OX} : Selectivity of ortho-xylene; Sel_{MX} : Selectivity of meta-xylene;

Sel_{PX} : Selectivity of para-xylene.

Figures and Tables

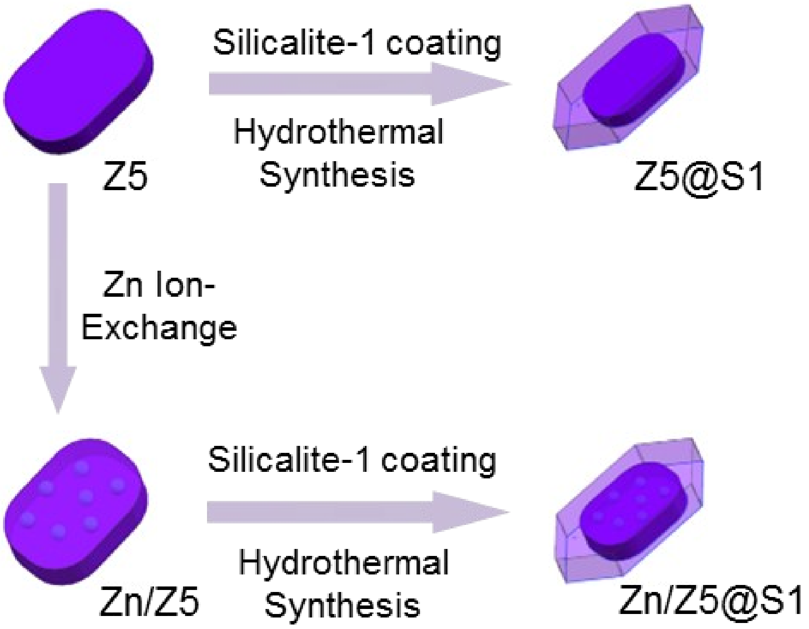

Scheme S1 Illustration on the synthesis of the Zn/Z5 and the typical core-shell-structured Z5@S1 and Zn/Z5@S1 zeolite with Z5 (Si/Al=46) as based zeolite.

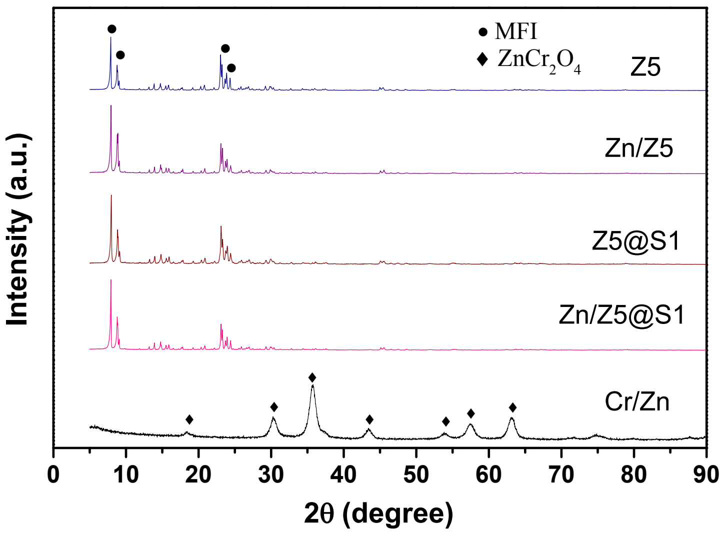

Figure S1. XRD patterns of the Z5, Zn/Z5, Z5@S1, Zn/Z5@S1 and Cr/Zn catalysts.

The XRD profiles of all the zeolite components used for hybrid catalyst preparation are shown in Figure S1.

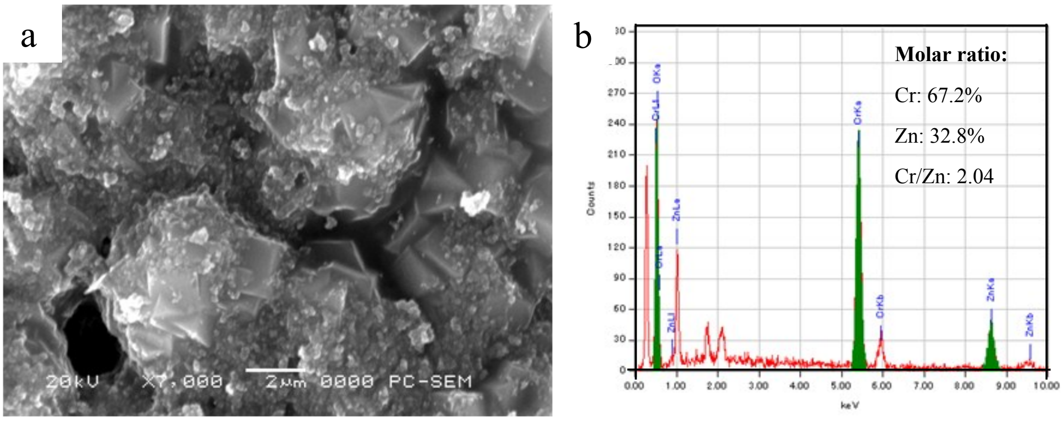

Figure S2. The (a) SEM image and (b) EDS analysis results of the Cr/Zn catalyst.

The physical morphology and element composition of Cr/Zn catalyst were determined by SEM-EDS. Figure S2 presented the SEM and EDS analysis results, in which the molar ratio of Cr/Zn is 2.04.

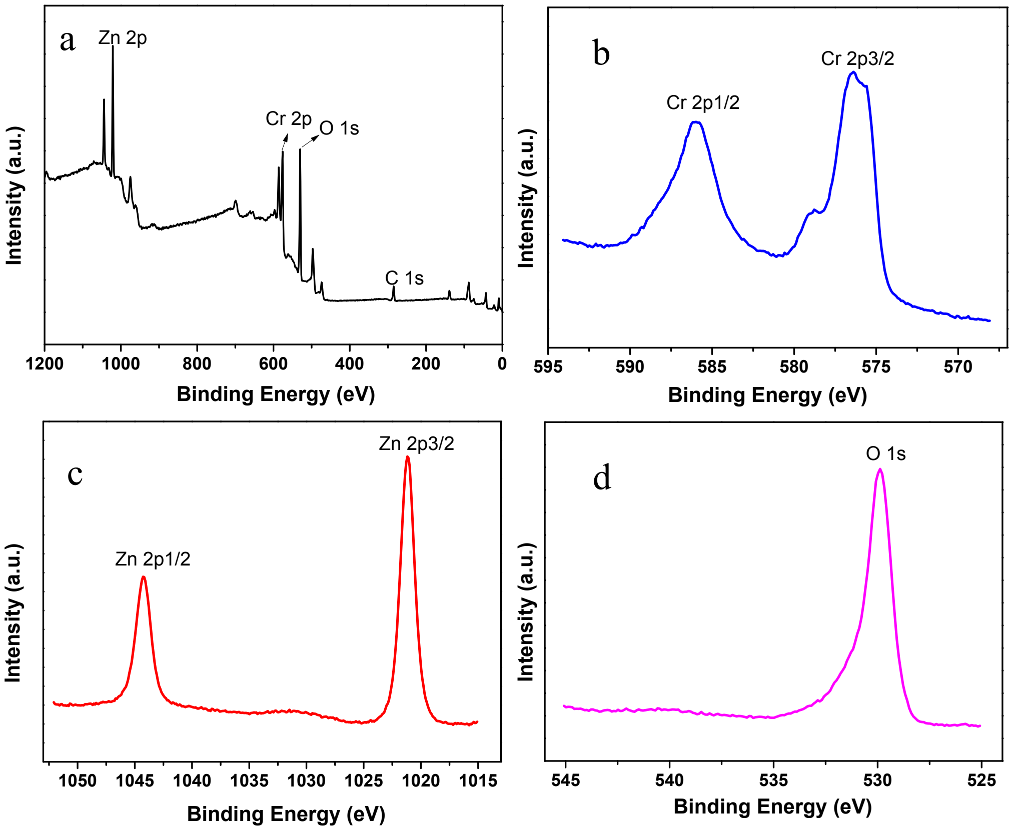

Figure S3. XPS spectra of Cr/Zn catalyst: (a) XPS survey spectrum, (b) high-resolution Cr 2p, (c) high-resolution Zn 2p, (d) high-resolution O 1s.

XPS was used to determine the chemical composition and electronic properties of Cr/Zn catalyst in the near-surface regions. XPS spectra of Cr/Zn with survey spectrum and high-resolution of Cr, Zn and O were illustrated in Figure S3. The primary spectra of Zn 2p, Cr 2p, O 1s and C 1s were obtained from the XPS survey of Cr/Zn oxides (Figure S3a). Moreover, the ratio of Cr/Zn was 2.08 from XPS, which was in agreement with XRD and EDS results.

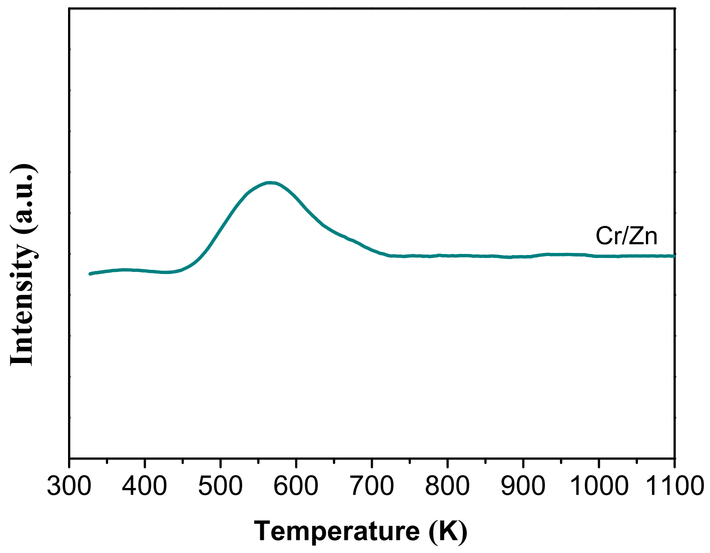

Figure S4. H_2-TPR profile of Cr/Zn catalyst.

The reducibility of Cr/Zn catalyst was evaluated by H_2-TPR and the profile was shown in Figure S4. The Cr/Zn catalyst presented one main hydrogen consumption peak located in the range of 500-673 K.

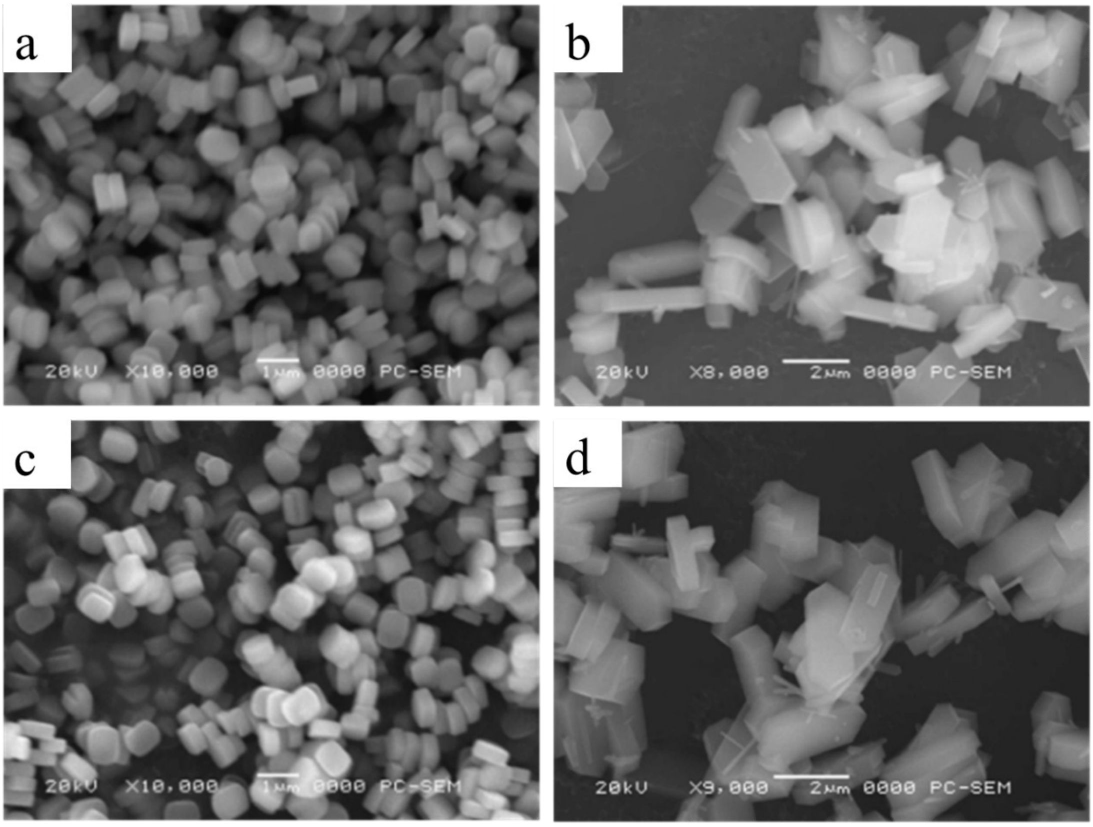

Figure S5. SEM images of a) Z5, b) Z5@S1, c) Zn/Z5 and d) Zn/Z5@S1 materials.

We employed SEM to characterize all the zeolite catalysts and the results were shown in Figure S5.

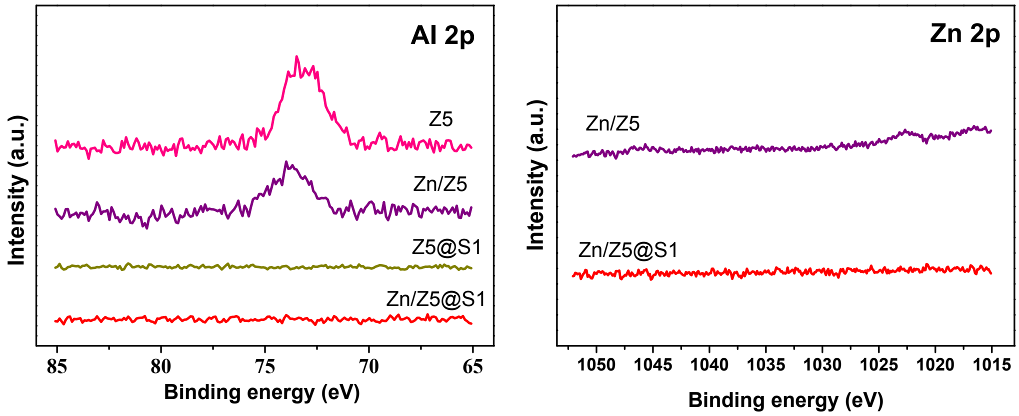

Figure S6. The XPS curves of Al and Zn on the zeolite components Z5, Zn/Z5, Z5@S1 and Zn/Z5@S1.

Figure S6 showed the XPS spectrum of Al species on the Z5, Zn/Z5, Z5@S1, Zn/Z5@S1 and Zn species on the Zn/Z5, Zn/Z5@S1. The amounts of element in near-surface of zeolite were listed on Table S1. There was no Al signal at the location of 73~74 eV on the Z5@S1 and Zn/Z5@S1 samples, implying that no Al existed on the surface of Z5@S1 and Zn/Z5@S1 samples and the epitaxial Silicalite-1 shell encapsulated the inner Z5 and Zn/Z5 core zeolite well. Furthermore, although few Zn signals were observed on the Zn/Z5 samples, it was also not discovered on the Zn/Z5@S1 since the well synthesized Silicalite-1 shell.

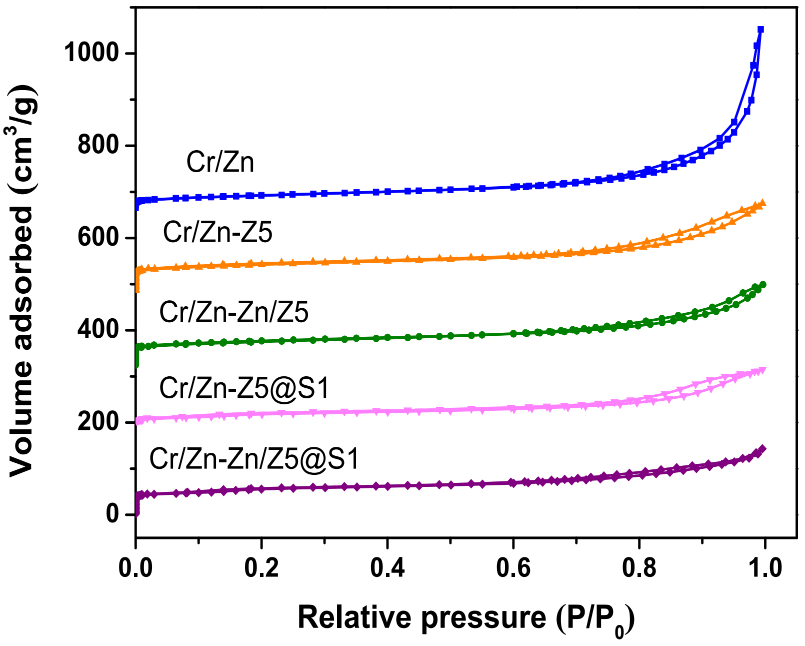

Figure S7. The N_2 adsorption-desorption isotherms of Cr/Zn, Cr/Zn-Z5, Cr/Zn-Zn/Z5, Cr/Zn-Z5@S1 and Cr/Zn-Zn/Z5@S1 catalysts.

The textural properties of different catalysts were measured by nitrogen physical adsorption. The nitrogen adsorption-desorption isotherms of different catalysts were shown in Figure S7. The corresponding physical properties, BET surface area, pore volume and size, were listed in the followed Table S2.

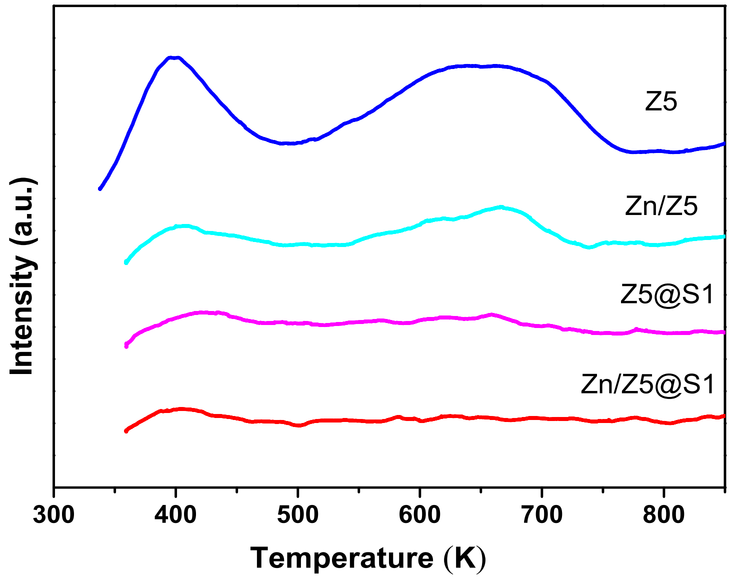

Figure S8. The NH_3-TPD curves of Z5, Zn/Z5, Z5@S1 and Zn/Z5@S1.

The acidity properties of the zeolite components used for hybrid catalysts preparation were evaluated by NH_3-TPD, and the results were shown in Figure S8 and Table S3. The NH_3 desorption temperature demonstrated the acidic strength, and the peak area revealed the acidic concentration of these zeolite components. It was clear that all the samples presented two desorption peaks: the first peak below 470 K corresponding to weak acid sites; the second peak above 570 K assigning with strong acid sites. The Z5@S1 and Zn/Z5@S1 had a lower acidic strength and acidic concentration than Z5 and Zn/Z5. The neutral properties of Silicalite-1 shell neutralized the total acidic properties of ZSM-5 zeolite.

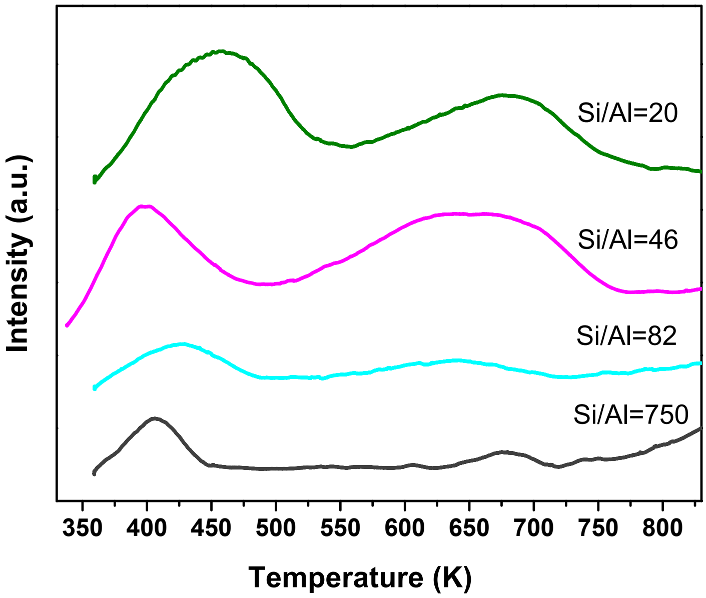

Figure S9. The NH_3-TPD curves of Z5 zeolite with different Si/Al ratio of 20, 46, 82 and 750.

The NH_3-TPD profiles of H-ZSM-5 with different Si/Al ratio were displayed in the Figure S9. The NH_3 desorption peak areas of weak and strong acid sites decreased with increasing the Si/Al ratio. It was consistent with the acidic sites from the calculation, as presented in Table S5.

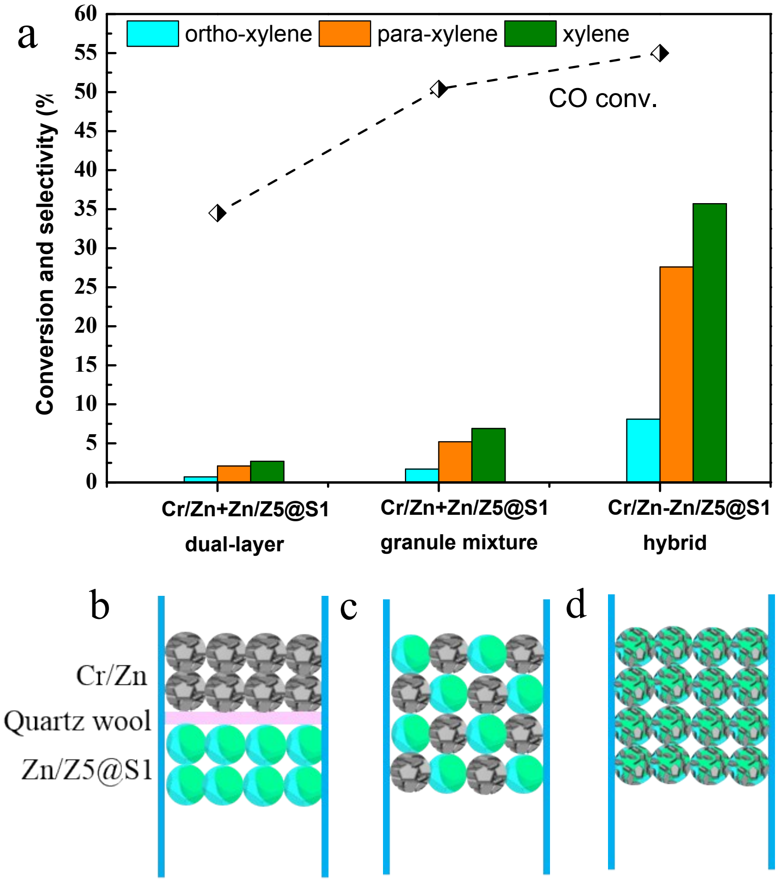

Figure S10. The effect of varied assembly models of two active components on the catalytic performance in reaction

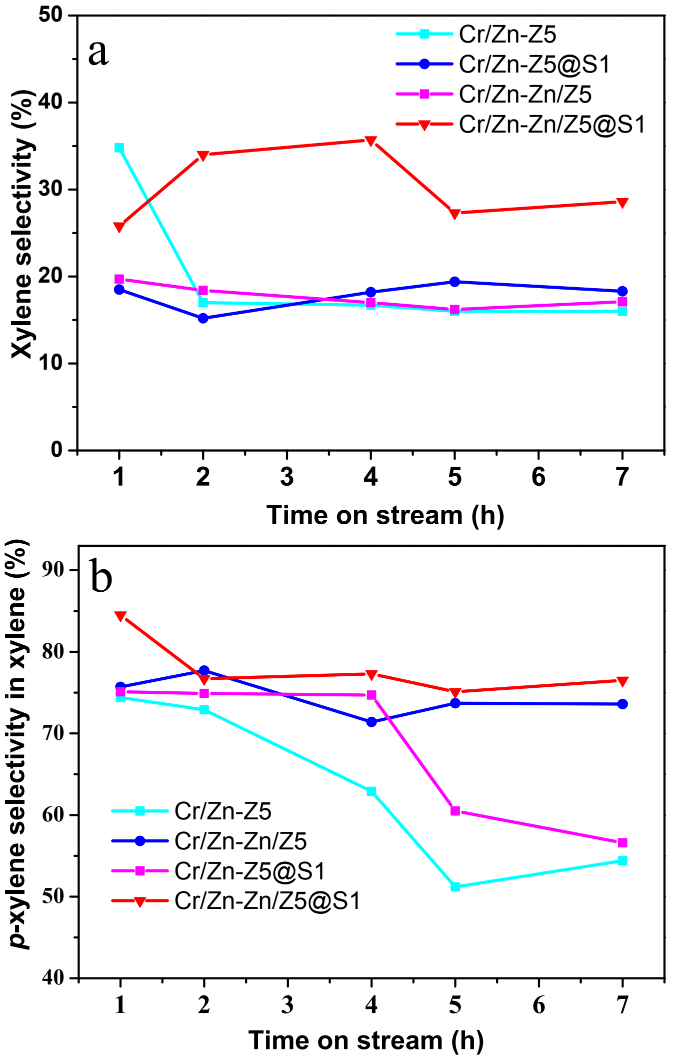

Figure S11. The xylene selectivity (a) and the p-xylene selectivity in the total xylene isomers (b) as a function of time on stream.

Time on stream of xylene and PX selectivities in the xylene isomers is shown in Figure S11. The Cr/Zn-Zn/Z5@S1 hybrid catalyst demonstrated the enhanced xylene selectivity and excellent stability of PX selectivity in xylene isomers during total reaction time.

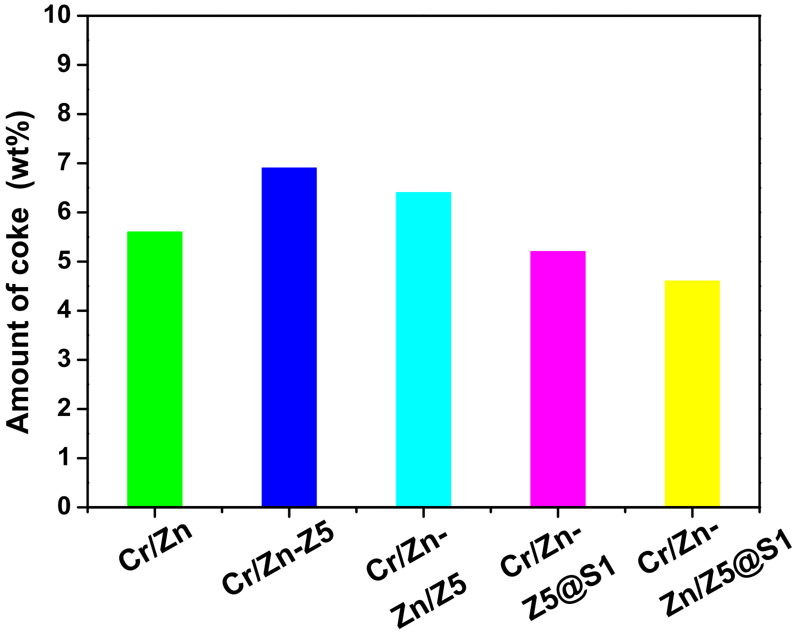

Figure S12. The coke deposition amount on the spent hybrid catalysts measured by TG.

TG was applied to measure the deposited coke on the spent hybrid catalysts. The analysis results of the spent catalysts after 10 h reaction time were compared in Figure S12.

Table S1 The atom amount of Si, Al, Zn and the atom ratio of Si/Al, Zn/Al on the zeolite components obtained from the XPS analysis results

Samples	Si	Al	Zn	Si/Al	Zn/Al
Z5	30.6	0.21	-	145.7	-
Zn/Z5	27.6	0.17	0.08	162.4	0.47
Z5@S1	36.6	0	-	-	-
Zn/Z5@S1	29.2	0	0	-	-

Table S2 BET surface area, pore volume and average pore size of samples.

Catalysts	S_{BET} (m^2g^{-1})	Pore volume (cm^3g^{-1})	Average pore size (nm)
Cr/Zn	98.9	0.58	21.5
Cr/Zn-Z5	188.4	0.26	5.8
Cr/Zn-Zn/Z5	190.8	0.28	5.9
Cr/Zn-Z5@S1	163.5	0.22	5.5
Cr/Zn-Zn/Z5@S1	186.7	0.21	4.4
Z5	370.6	0.23	0.58
Zn/Z5	382.1	0.19	0.58
Z5@S1	363.1	0.22	0.58
Zn/Z5@S1	383.3	0.20	0.58

As presented in Table S2, Cr/Zn catalysts illustrated BET surface area of 98.9 m^2/g, pore volume of 0.58 cm^3/g and 21.5 nm averaged pore size. The BET surface area, pore volume and average pore size of hybrid catalysts were similar because of the same ratio of Cr/Zn and approximate textural properties of Z5-based zeolite materials.

Table S3 The acidity properties of different zeolite components used for hybrid catalysts preparation.

Samples	Total amount (mmol/g)	Amount (mmol/g)	
		Weak acid sites	Strong acid sites
Z5	0.36	0.12	0.24
Zn/Z5	0.27	0.09	0.18
Z5@S1	0.15	0.07	0.08
Zn/Z5@S1	0.09	0.04	0.05

Table S4 The acidity properties of Z5 zeolite characterized by NH_3-TPD.

Si/Al ratio	Total amount (mmol/g)	Amount (mmol/g)	
		Weak acid sites	Strong acid sites
20	0.67	0.35	0.32
46	0.36	0.12	0.24
82	0.19	0.10	0.09
750	0.12	0.08	0.04

Table S5 The one pass PX synthesis from syngas over the simplest hybrid catalyst of Cr/Zn-Z5 with varied Si/Al ratio[a]

Catalysts	Si/Al	Conv. (%)		Sel. (%)[b]											
		CO	CO_2	C_1	C_2	C_3	C_4	C_5	C_6	C_7	C_8	OX	MX	PX	Others[c]
Cr/Zn-Z5	20	62.8	-57.2	9.0	15.3	38.7	8.9	2.2	0.3	14.0	4.9	3.0	0	3.0	0.7
	46	61.0	-48.0	2.4	6.7	12.3	12.3	9.2	4.3	10.0	25.2	6.1	0.1	10.5	0.9
	82	58.0	-46.5	9.3	18.2	19.3	10.9	7.1	1.9	2.9	12.2	5.8	0.1	9.8	2.5
	750	53.8	-46.7	8.3	11.1	10.1	5.6	4.9	5.7	7.3	32.3	5.3	0.5	8.6	0.3

[a] Reaction conditions: 5.0 MPa, 673 K, W/F=20.7 g·h·mol^{-1}, 4 h, 0.5 g catalyst, Cr/Zn=2/1, the weight ratio of Cr/Zn to Z5 was 2, syngas $H_2/CO/CO_2/Ar$=62.78/29.3/4.95/2.97. [b] ortho-xylene=OX, meta-xylene=MX, para-xylene=PX, C_6-C_8 are the aromatics except for xylene. [c] Others mainly consisted of ethene and propene.

We prepared a series of hybrid catalysts Cr/Zn-Z5 with the Z5 zeolite that has varied Si/Al ratio as zeolite component. We investigated their catalytic performance for one-pass PX synthesis from syngas. The reaction results were shown in Table S5. The CO conversion decreased slightly with increasing the Si/Al ratio of Z5. It seems that the Z5 zeolite with Si/Al ratio of 46 was more suitable for PX synthesis over the hybrid catalyst. Therefore, other hybrid catalysts were prepared by mixing Cr/Zn and the Z5 (Si/Al=46) derived zeolite components.

Table S6 The catalytic performance of varied catalysts for the one-pass PX synthesis from syngas[a]

Catalysts	Conv. (%)		Sel. (%)[b]													
	CO	CO_2	MeOH	DME	C_1	C_2	C_3	C_4	C_5	C_6	C_7	C_8	OX	MX	PX	others[c]
Cr/Zn	27.5	-27.5	15.9	38.2	30.4	8.5	0.5	2.2	2.8	1.1	0.4	0	0	0	0	0
Cr/Zn-Z5	61.0	-61.0	0	0	2.4	6.7	12.3	12.3	9.2	4.3	10.0	25.2	6.1	0.1	10.5	0.9
Cr/Zn-Z5@S1	55.2	-55.2	0	0	2.4	14.4	14.9	7.5	5.8	6.8	6.8	21.3	4.9	0.3	13.0	1.9
Cr/Zn-Zn/Z5	66.4	-66.4	c	0	3.2	6.0	10.1	12.5	9.3	2.4	9.6	29.7	3.9	0.3	12.4	0.6
Cr/Zn-Zn/Z5@S1 hybrid	55.0	-55.0	0	0	4.4	13.7	10.9	5.1	3.7	3.6	9.5	13.2	8.1	0	27.6	0.2
Cr/Zn+Zn/Z5@S1 dual-layer	34.5	8.0	0.2	0.1	26.8	10.7	11.2	20.0	15.5	8.3	1.2	1.5	0.7	0	2.1	1.7
Cr/Zn+Zn/Z5@S1 granule mixture	50.4	-29.8	0.1	0	7.2	12.0	14.2	20.5	19.3	14.8	3.2	1.2	1.7	0	5.2	0.6

[a] Reaction conditions: 5.0 MPa, 673 K, W/F=20.7 g·h·mol^{-1}, 4 h, 0.5 g catalyst, Cr/Zn=2/1, the weight ratio of Cr/Zn to zeolite was 2, syngas $H_2/CO/CO_2/Ar$=62.78/29.3/4.95/2.97. [b] ortho-xylene=OX, meta-xylene=MX, para-xylene=PX, C_6-C_8 are the aromatics except for xylene. [c] Others mainly consisted of ethene and propene.

The Cr/Zn catalyst and all hybrid catalysts were evaluated to realize the PX direct synthesis from syngas and the reaction results were shown in Table S6. The Cr/Zn-Zn/Z5@S1 hybrid catalyst presented excellent para-xylene selectivity of 27.6% with CO conversion of 55.0%.

Table S7 The catalytic performance of Cr/Zn-Zn/Z5@S1 hybrid catalyst prepared with varied weight ratio of Cr/Zn to Zn/Z5@S1 for the one-pass of PX synthesis from syngas[a]

Catalysts	Conv. (%)		Sel. (%)[b]											
	CO	CO_2	C_1	C_2	C_3	C_4	C_5	C_6	C_7	C_8	OX	MX	PX	others[c]
Cr/Zn-Zn/Z5@S1-2/1	55.0	-55.0	4.4	13.7	10.9	5.1	3.7	3.6	9.5	13.2	8.1	0	27.6	0.2
Cr/Zn-Zn/Z5@S1-1/1	54.6	-46.6	3.0	5.7	8.1	8.7	8.4	5.3	7.8	28.7	6.5	0.7	16.5	0.6
Cr/Zn-Zn/Z5@S1-1/2	47.3	-36.4	2.4	4.9	6.7	7.9	8.0	4.6	6.6	35.0	5.9	0.2	17.1	0.7

[a] Reaction conditions: 5.0 MPa, 673 K, W/F=20.7 g·h·mol^{-1}, 4 h, 0.5 g catalyst, Cr/Zn=2/1, syngas $H_2/CO/CO_2/Ar$=62.78/29.3/4.95/2.97. [b] ortho-xylene=OX, meta-xylene=MX, para-xylene=PX, C_6-C_8 are the aromatics except for xylene. [c] Others mainly consisted of ethene and propene.

We prepared a series of hybrid catalysts Cr/Zn-Zn/Z5@S1 with different weight ratios of Cr/Zn and Zn/Z5@S1. We investigated their catalytic performance for one-pass PX synthesis from syngas. The reaction results were shown in Table S7. The CO conversion decreased slightly with increasing the Zn/Z5@S1 amount. Moreover, the Cr/Zn-Zn/Z5@S1-2/1 realized the highest PX selectivity of 27.6%. It confirmed that the Cr/Zn zeolite with Zn/Z5@S1 ratio of 2 was more suitable for PX synthesis over the hybrid catalyst.
